# Autophagy-Related Gene 7 Polymorphisms and Cerebral Palsy in Chinese Infants

**DOI:** 10.3389/fncel.2019.00494

**Published:** 2019-11-05

**Authors:** Lei Xia, Jianhua Xu, Juan Song, Yiran Xu, Bohao Zhang, Chao Gao, Dengna Zhu, Chongchen Zhou, Dan Bi, Yangong Wang, Xiaoli Zhang, Qing Shang, Yimeng Qiao, Xiaoyang Wang, Qinghe Xing, Changlian Zhu

**Affiliations:** ^1^Henan Key Laboratory of Child Brain Injury, Institute of Neuroscience and the Third Affiliated Hospital of Zhengzhou University, Zhengzhou, China; ^2^Institutes of Biomedical Sciences and Children’s Hospital, NHC Key Lab of Reproduction Regulation, Fudan University, Shanghai, China; ^3^Child Rehabilitation Center, Children’s Hospital Affiliated to Zhengzhou University, Zhengzhou, China; ^4^Child Rehabilitation Center, The Third Affiliated Hospital of Zhengzhou University, Zhengzhou, China; ^5^Henan Key Laboratory of Child Inherited Metabolic Disease, Children’s Hospital Affiliated to Zhengzhou University, Zhengzhou, China; ^6^Center for Perinatal Medicine and Helath, Sahlgrenska Academy, Gothenburg University, Gothenburg, Sweden; ^7^Shanghai Center for Women and Children’s Health, Shanghai, China; ^8^Center for Brain Repair and Rehabilitation, Sahlgrenska Academy, Gothenburg University, Gothenburg, Sweden

**Keywords:** autophagy, *ATG7*, cerebral palsy, luciferase reporter gene, single nucleotide polymorphisms

## Abstract

Cerebral palsy (CP) is a group of non-progressive motor impairment syndromes that are secondary to brain injury in the early stages of brain development. Numerous etiologies and risk factors of CP have been reported, and genetic contributions have recently been identified. Autophagy has an important role in brain development and pathological process, and autophagy-related gene 7 (*ATG7*) is essential for autophagosome biogenesis. The purpose of this study was to investigate the genetic association between *ATG7* gene single nucleotide polymorphisms (SNPs) and CP in Han Chinese children. Six SNPs (rs346078, rs1470612, rs11706903, rs2606750, rs2594972, and rs4684787) were genotyped in 715 CP patients and 658 healthy controls using the MassArray platform. Plasma ATG7 protein was determined in 73 CP patients and 79 healthy controls. The differences in the allele and genotype frequencies of the rs1470612 and rs2594972 SNPs were determined between the CP patients and controls (*p*_*allele*_ = 0.02 and 0.0004, *p*_*genotype*_ = 0.044 and 0.0012, respectively). Subgroup analysis revealed a more significant association of rs1470612 (*p*_*allele*_ = 0.004, *p*_*genotype*_ = 0.0036) and rs2594972 (*p*_*allele*_ = 0.0004, *p*_*genotype*_ < 0.0001) with male CP, and more significant differences in allele and genotype frequencies were also noticed between CP patients with spastic diplegia and controls for rs1470612 (*p*_*allele*_ = 0.0024, *p_*genotype*_* = 0.008) and rs2594972 (*p*_*allele*_ < 0.0001, *p_*genotype*_* = 0.006). The plasma ATG7 level was higher in CP patients compared to the controls (10.58 ± 0.85 vs. 8.18 ± 0.64 pg/mL, *p* = 0.024). The luciferase reporter gene assay showed that the T allele of rs2594972 SNP could significantly increase transcriptional activity of the ATG7 promoter compared to the C allele (*p* = 0.009). These findings suggest that an association exists between genetic variants of *ATG7* and susceptibility to CP, which provides novel evidence for the role of ATG7 in CP and contributes to our understanding of the molecular mechanisms of this neurodevelopmental disorder.

## Introduction

Cerebral palsy (CP) is a group of permanent neurological disabilities that manifest during early childhood and affect movement and posture, with a prevalence of 2–3/1000 live births (MacLennan et al., 2015; Nelson and Blair, 2015; Lim, 2016). CP is a static neurological condition resulting from brain injury in the perinatal and neonatal periods, and the motor disorders of CP are often accompanied by epilepsy, secondary musculoskeletal problems, and disturbances in sensation, perception, cognition, communication, and behavior. There are a number of risk factors for CP, including asphyxia, prematurity, and intrauterine infection (Zhu et al., 2009; Lee et al., 2015; Song et al., 2016; Zhang et al., 2017). However, several studies have shown that many children with one or more of these risk factors do not develop CP (Zhu et al., 2009; Song et al., 2016; Korzeniewski et al., 2018), and there is evidence for genetic susceptibility to CP, but little is known about possible mechanisms for this (O’Callaghan et al., 2011; Moreno-De-Luca et al., 2012; MacLennan et al., 2018). Although a number of genes and genetic loci have been shown to be associated with CP (Bi et al., 2014; Xia et al., 2018; Sun et al., 2019), the genetic causes and underlying molecular mechanisms behind sporadic CP remain largely unknown (Fahey et al., 2017; MacLennan et al., 2019).

Autophagy is a highly conserved cellular process that delivers damaged proteins and organelles to lysosomes for digestion (Rubinsztein et al., 2012), and autophagy is involved in intracellular quality control, starvation adaptation, development and aging (Oppenheim et al., 2008). It plays an important role in the process of normal brain development and defense against pathological processes (Komatsu et al., 2006; Oppenheim et al., 2008; Yamamoto and Yue, 2014), and autophagy deficiency or excessive activation can induce cell death and lead to pathological conditions (Zhu et al., 2006; Xie et al., 2016). Autophagy is carried out through the activity of multiple autophagy-related genes (*Atg*). More than 30 *Atg* genes have been identified in yeast, most of which are conserved in mammals (Mizushima, 2007). *Atg7* is essential for autophagy, and depletion of *Atg7* suppresses autophagy in mice (Komatsu et al., 2006; Xie et al., 2016). It has been shown that *Atg7* plays a vital role in the development of axons and dendrites and in synapse maturation processes (Kim et al., 2016), and selective deletion of *Atg7* revealed its roles in adipogenesis, gluconeogenesis, and lipid metabolism (Rubio-Gonzalez et al., 2015).

A clinical study found *ATG7* variants in neurological diseases (Chen et al., 2013), and it has been suggested that changes in ATG7 protein expression and polymorphisms in *ATG* genes are associated with aberrant autophagy activity and contribute to the pathological processes of these diseases (Pang et al., 2012; Chen et al., 2013; Agler et al., 2014; Xie et al., 2016; Wang et al., 2017; Xu et al., 2017). In addition, we and others have shown that autophagy activity is increased in the immature brain after hypoxia ischemia insult (Ginet et al., 2014a; Sun et al., 2016), and selective *Atg7* knockout significantly reduces neuronal cell death and brain injury in neonatal mice after hypoxia ischemia (Koike et al., 2008; Xie et al., 2016).

Despite all of the work described above, there are no reports yet regarding *Atg7* gene variants and CP, which is the most common motor function disability among children. Based on our previous studies on CP genetics and autophagy in the developing brain, the current study sought to investigate the possible association of *ATG7* single nucleotide polymorphisms (SNPs) and ATG7 protein expression in the blood with susceptibility to CP in a Han Chinese child cohort.

## Materials and Methods

### Subjects

This study was a Han Chinese population-based case-control study. Sporadic CP patients were from the Department of Children’s Rehabilitation, the Third Affiliated Hospital of Zhengzhou University, and Children’s Hospital of Zhengzhou University, China. The sporadic CP cases (*n* = 715) had a mean age of 18.26 ± 15.13 months and included 219 girls and 496 boys, 675 term and 40 preterm infants, 468 cases with spastic CP and 247 cases with non-spastic CP, 686 cases with birth body weight ≥ 2,500 g and 29 cases with birth body weight < 2,500 g, 212 cases with asphyxia and 503 without asphyxia, 67 cases with periventricular leukomalacia and 648 cases without periventricular leukomalacia, and 289 cases of comorbidity with intellectual disability and 426 cases without intellectual disability. Among the spastic CP cases, there were 270 cases of quadriplegic CP, 121 cases of diplegic CP, 48 cases of hemiplegic CP, 12 cases of monoplegic CP, and 17 cases of other types. Ethnic-matched healthy controls (*n* = 658) were from Department of Child Healthcare in the same hospital, and these controls had a mean age of 19.53 ± 17.18 months and included 220 girls and 438 boys, 647 term and 11 preterm infants, 641 cases with birth body weight ≥ 2,500 g and 17 cases with birth body weight < 2,500 g, and 10 cases with asphyxia and 648 cases without asphyxia (Table 1). A total of 73 CP patients (23 girls and 50 boys, 65 term and 8 preterm infants, 56 cases with spastic CP and 17 cases with non-spastic CP, 67 cases with birth body weight ≥ 2,500 g and 6 cases with birth body weight < 2,500 g, 17 cases with asphyxia and 56 cases without asphyxia, and 30 cases of comorbidity with intellectual disability and 43 cases without intellectual disability) and 79 healthy controls (19 girls and 60 boys, 77 term and 2 preterm infants, 79 cases with birth body weight ≥ 2,500 g, and 2 cases with asphyxia and 77 cases without asphyxia) were selected for the plasma ATG7 protein assay. All CP patients were diagnosed by two child neurologists according to the guidelines proposed by the “Surveillance of CP in Europe” network. Controls with any neurologic condition (seizure disorder, attention-deficit/hyperactivity disorder, developmental delay, or migraine headache) or pre-defined medical conditions (juvenile diabetes mellitus) or with a family history of nervous system diseases were excluded. All subjects were Han Chinese from the Henan Province, and informed consent to participate in this study was approved by the children’s parents. This study was approved by the Ethics Committee of Zhengzhou University (201201002) in accordance with the Helsinki Declaration.

**TABLE 1:** Sample description for gene polymorphism analysis.

**Characteristic**	**CP cases**		**Control**	
	**Total (%)**	**M/F (n)**	**Total (%)**	**M/F (n)**
**Gestational age**				
Preterm (<37 weeks)	40 (5.59)	33/7	11 (1.67)	10/1
Term (≥37 weeks)	675 (94.41)	463/212	647 (98.33)	428/219
Total	715 (100)	496/219	658 (100)	438/220
**Type of CP**				
Spastic CP	468 (65.45)	338/130	−	–
Non-spastic CP	247 (34.55)	158/89	−	–
Total	715 (100)	496/219	658 (100)	438/220
**Birth weight**				
<2500 g	29 (4.06)	20/9	17 (2.58)	13/4
≥2500 g	686 (95.94)	485/201	641 (97.42)	425/216
Total	715 (100)	505/210	658 (100)	438/220
**Birth asphyxia**				
Asphyxia	212 (29.65)	157/55	10 (1.52)	7/3
No Asphyxia	503 (70.35)	339/164	648 (98.48)	431/217
Total	715 (100)	496/219	658 (100)	438/220
**Maternal factors**				
PROM	68 (951)	51/17	24 (3.65)	16/8
No PROM	647 (90.49)	425/202	634 (96.35)	420/214
TPL	57 (7.97)	40/17	33 (5.02)	27/6
No TPL	648 (92.03)	454/196	625 (94.98)	411/214
PIH	27 (3.78)	21/6	8 (1.22)	7/1
No PIH	688 (96.22)	476/212	650 (98.78)	431/219
**Complication**				
With PVL	67 (9.37)	53/14		
Without PVL	648 (90.63)	445/203		
With HIE	96 (13.45)	71/25		
Without HIE	619 (86.55)	421/198		
With MR	289 (40.42)	199/90		
Without MR	426 (59.58)	297/129		

*CP, cerebral palsy; M, male; F, female; PVL, periventricular leukomalacia; PROM, premature rupture of membrane; TPL, threatened premature labor; PIH, pregnancy-induced hypertension; HIE, hypoxic ischemic encephalopathy; MR, mental retardation.*

### Sample Collection

The blood samples with heparin sodium with anti-coagulant were routinely used in the present study. Plasma was separated by centrifugation within 2 h after being collected and samples with hemolysis were excluded. DNA was extracted from the remaining blood components, and all of the prepared samples were stored at −80°C until use.

### Genotyping

A total of six SNPs (rs346078, rs1470612, rs11706903, rs2606750, rs2594972, rs4684787) of the *ATG7* gene whose minor allele frequencies in the Han Chinese population are greater than 0.1 were selected from the dbSNP database^1^. SNP Function Prediction^2^ showed that rs1470612 might be a transcription factor binding site, and a genome-wide association study reported that rs11706903 is associated with multiple sclerosis (Dang et al., 2016). All six SNPs were located in introns – rs346078 (3^rd^ intron), rs1470612 (4^th^ intron), rs11706903 (6^th^ intron), rs2606750 (10^th^ intron), rs2594972 (15^th^ intron), and rs4684787 (27^th^ intron).

After collecting the plasma, genomic DNA was isolated from blood leukocytes with the AxyPrep Blood Genomic DNA Miniprep Kit (Axygen Biosciences, Union City, CA, United States) according to the instructions of the kit. After the amplification of SNP-spanning fragments by multiplex PCR, SNP genotyping was performed using the Sequenom iPlex MassARRAY platform according to the instructions (Sequenom, San Diego, CA, United States). The probes and primers were designed using the SEQUENOM online tools. The person who analyzed the genotype results was blinded to the clinical data of the subjects.

### ELISA for Plasma ATG7

The frozen plasma samples were thawed completely at room temperature, mixed well by vortexing, and centrifuged to collect the supernatants for the ATG7 protein determination. The ATG7 levels in the plasma of 73 patients and 79 controls were measured using a Human ATG7 ELISA Kit (LifeSpan BioSciences, Inc., Cat# LS-F9291) with a detection range of 0.313–20 ng/ml. All samples were diluted twofold based on preliminary tests with different dilutions, and the assay was performed according to the manufacturer’s protocol. The absorbance at 405 nm was measured with a SpectraMax 190 Microplate Spectrophotometer. The ATG7 level in plasma was calculated based on the kit standard, and the data were expressed as the mean ± SEM.

### Luciferase Reporter Assay

Luciferase reporter assays were performed to determine if the SNP influenced gene expression. The PrimeSTAR HS DNA polymerase (Takara) was used to amplify DNA fragments of interest, including the *ATG7* promoter (2040 bp), rs2594972 mutant, and wild-type (2241 bp), as recommended by the manufacturer. The *ATG7* promoter DNA fragment was inserted into a pGL3-promoterless luciferase-based plasmid’s (Shanghai Genechem, Co., Ltd.) multiple cloning site with *Kpn*I/*Xho*I to generate an ATG7-luciferase-construct (promoter only) reporter plasmid. The insertion was upstream of the Luciferase gene (luc +) at the promoter site. The ATG7 SNP sequences encompassing the SNP allele were individually inserted into the enhancer site of the ATG7-luciferase construct. These constructs were then transformed into *Escherichia coli* cells, and the cloned plasmids were amplified and verified by DNA sequencing. For the dual-luciferase reporter assay, 293T cells were plated onto 24-well plates and transfected with 300 ng of pGL3 or pGL3-ATG7-promoter with wild-type (C) or mutant rs2594972 (T) using X-tremegene (Roche). The experiment was triplicated, and luciferase activity was assessed 48 h after transfection using the dual-luciferase reporter assay system (Promega). Fold increase was calculated by Firefly luciferase activity normalized to Renilla luciferase activity.

### Statistical Analysis

All the analyses, including comparison of allele and genotype frequencies, Hardy–Weinberg equilibrium tests, and haplotype association analyses, calculation of odd ratios (ORs) and 95% confidence interval (95% CIs), estimation of pairwise linkage disequilibrium (LD), were performed on the SHEsis online software platform^3^. The program SNPSpD was used to correct for multiple testing performed on each individual SNP. Student’s unpaired *t*-test was used for the analysis of protein levels. Data with unequal variances were analyzed using the Mann–Whitney *U*-test. The Statistical Package for the Social Sciences (SPSS version 21.0) and GraphPad Prism 6.0 software package (version 6.0 for Windows, GraphPad, La Jolla, CA, United States) were used for statistical analyses. All reported *p*-values were two-tailed, and *p* < 0.05 was recognized as statistical difference.

## Results

### Overall Analysis of Gene Polymorphisms

The genotypic distribution of the six SNPs did not depart from Hardy–Weinberg equilibrium in the control group. We performed an association analysis using CP case-control groups, and significant differences were observed in both allele and genotype frequencies between the total CP patients (*n* = 715) and controls (*n* = 658) for rs1470612 (OR = 1.281, 95% CI = 1.077–1.523, *p*_*allele*_ = 0.02, *p_*genotype*_* = 0.044 after SNPSpD correction) and rs2594972 (OR = 1.376, 95% CI = 1.168–1.618, *p*_*allele*_ = 0.0004, *p_*genotype*_* = 0.0012 after SNPSpD correction) (Table 2). For rs1470612, the CP group had a significantly higher allele A and AA genotype frequency compare to the control group. For rs2594972, the CP group had a significantly higher allele T and TT genotype frequency. These results indicated that patients carrying the rs1470612 AA or rs2594972 TT genotypes had an increased risk of CP compared with those with other genotypes. No differences in other loci were observed.

**TABLE 2:** Allele and genotype frequencies of *ATG7* in total CP patients and controls.

	**Group**	**Allele frequency**		***p-Value***	**OR [95% CI]**	**Genotype frequency**			***p-Value***	**H-W^∗^**
rs346078		C	G			C/C	C/G	G/G		
	CP	361 (0.281)	925 (0.719)	0.297	0.911 [0.764–1.086]	48 (0.075)	265 (0.412)	330 (0.513)	0.573	
	Control	339 (0.300)	791 (0.700)			48 (0.085)	243 (0.430)	274 (0.485)		0.568
rs1470612		A	G			A/A	A/G	G/G		
	CP	433 (0.341)	837 (0.659)	0.005^a^	1.281 [1.077–1.523]	79 (0.124)	275 (0.433)	281 (0.443)	0.011^b^	
	Control	324 (0.288)	802 (0.712)			43 (0.076)	238 (0.423)	282 (0.501)		0.457
rs11706903		A	C			A/A	A/C	C/C		
	CP	415 (0.330)	841 (0.670)	0.044	1.197 [1.005–1.425]	73 (0.116)	269 (0.428)	286 (0.455)	0.136	
	Control	327 (0.292)	793 (0.708)			50 (0.089)	227 (0.405)	283 (0.505)		0.644
rs2606750		A	G		1.076 [0.906–1.278]	A/A	A/G	G/G		
	CP	429 (0.383)	691 (0.617)	0.404		102 (0.182)	225 (0.402)	233 (0.416)	0.357	
	Control	401 (0.366)	695 (0.634)			83 (0.151)	235 (0.429)	230 (0.420)		0.076
rs2594972		T	C			T/T	C/T	C/C		
	CP	552 (0.461)	646 (0.539	0.0001^c^	1.376 [1.168–1.618]	153 (0.255)	246 (0.411)	200 (0.334)	0.0003^d^	
	Control	453 (0.383)	729 (0.617)			95 (0.161)	263 (0.445)	233 (0.394)		0.154
rs4684787		T	C			T/T	C/T	C/C		
	CP	466 (0.362)	822 (0.638)	0.475	0.941 [0.797–1.111]	77 (0.120)	312 (0.484)	255 (0.396)	0.720	
	Control	421 (0.376)	699 (0.624)			70 (0.125)	281 (0.502)	209 (0.373)		0.100

*^*a*^After the SNPSpD correction, the *p-*value is 0.020; ^*b*^After the SNPSpD correction, the *p-*value is 0.044; ^*c*^After the SNPSpD correction, the *p-*value is 0.0004; ^*d*^After the SNPSpD correction, the *p-*value is 0.0012. ^∗^Hardy–Weinberg equilibrium.*

D′ and r^2^ were calculated to clarify the extent of LD in pairwise combinations of the six SNPs. The pairwise D′ values are shown in Table 3. Strong LD among the four SNPs (rs346078, rs1470612, rs11706903, and rs2606750) was observed (D′ > 0.8).

**TABLE 3:** The linkage disequilibrium among the SNPs in *ATG7.*

**D**′**/r^2^**	**rs346078**	**rs1470612**	**rs11706903**	**rs2606750**	**rs2594972**	**rs4684787**
rs346078		0.956	0.992	0.898	0.565	0.646
rs1470612	0.174		0.978	0.844	0.902	0.779
rs11706903	0.185	0.937		0.866	0.917	0.821
rs2606750	0.210	0.536	0.548		0.731	0.422
rs2594972	0.093	0.564	0.584	0.528		0.562
rs4684787	0.290	0.165	0.181	0.064	0.129	

*The standardized D′ values are shown above the diagonal, and the *r*^2^ values are shown below the diagonal.*

In order to determine whether the SNPs would have greater predictive value when analyzed together, haplotypes containing the rs346078, rs1470612, rs11706903, and rs2606750 SNPs were analyzed. Haplotype analysis revealed 13 possible haplotypes, the most common being GGCG (32%) and CGCG (31%) (Table 4). Eight haplotypes had an estimated frequency below 3% in both controls and cases. A significant difference was identified in global haplotype distribution (*p* = 0.0004) between CP and controls, and the haplotype GAAG was more prevalent among patients than controls and thus posed a risk for CP (*p* = 0.000026).

**TABLE 4:** The haploid type analysis in *ATG7.*

**Haplotype**	**Case (freq)**	**Control (freq)**	***p-*Value**	**OR [95% CI]**
CGCG	277.40 (0.283)	285.91 (0.309)	0.271	0.895 [0.734–1.091]
GAAA	276.72 (0.282)	241.31 (0.261)	0.231	1.132 [0.924–1.387]
GGCA	74.45 (0.076)	75.84 (0.082)	0.674	0.931 [0.667–1.299]
GGCG	289.30 (0.295)	299.31 (0.324)	0.233	0.888 [0.730–1.080]
GAAG	36.22 (0.037)	7.64 (0.008)	0.00002	4.661 [2.125–10.226]
Global results			0.00044	

*Loci chosen for haplotype analysis: rs346078, rs1470612, rs11706903, and rs2606750. Haplotypes with a frequency < 0.03 in both controls and cases have been dropped. OR, odds ratio; CI, confidence interval.*

### Subgroup Analysis

The phenotype and etiology of CP are highly heterogeneous. The further subgroup analysis of SNPs was done according to gestational age, sex, birth weight, perinatal asphyxia, subtype of CP, maternal factors, and pregnancy complications. Compared to the association analysis of global CP, more significant differences in allele and genotype frequencies were noticed between CP patients (*n* = 496) and controls (*n* = 438) in males for rs11706903 (OR = 1.318, 95% CI = 1.067–1.628, *p_*allele*_* = 0.040), rs1470612 (OR = 1.408, 95% CI = 1.141–1.738, *p*_*allele*_ = 0.004, *p_*genotype*_* = 0.0036), and rs2594972 (OR = 1.515, 95% CI = 1.244–1.848, *p*_*genotype*_ < 0.0001) (Table 5). In addition, more significant differences in allele and genotype frequencies were observed between CP patients with spastic diplegia (*n* = 121) and controls (*n* = 658) for rs1470612 (OR = 1.689, 95% CI = 1.249–2.287, *p*_*allele*_ = 0.0024, *p_*genotype*_* = 0.008) and rs2594972 (OR = 1.718, 95% CI = 1.236–2.387, *p*_*allele*_ < 0.0001) (Table 6). Further analysis of the allele and genotype distribution of the SNPs in patients with or without maternal premature rupture of membrane showed a significant difference in allele frequencies between CP patients with premature rupture of membrane (*n* = 68) and controls (*n* = 658) for rs11706903 (OR = 1.480, 95% CI = 1.007–2.175, *p*_*allele*_ = 0.045) and rs1470612 (OR = 1.511, 95% CI = 1.028–2.221, *p*_*allele*_ = 0.035). However, these significant associations disappeared after Bonferroni correction for multiple testing. The differences in allele and genotype frequencies were not significant between CP patients with periventricular leukomalacia, neonatal asphyxia, pregnancy complications, or maternal factors and control subjects for any of the six SNPs of *ATG7*.

**TABLE 5:** Allele and genotype frequencies of *ATG7* in male CP patient and male controls.

**Gene**	**Group**	**Allele frequency**		***p*-Value**	**OR [95% CI]**	**Genotype frequency**			***p-*Value**	**H-W^∗^**
rs346078		C	G			C/C	C/G	G/G		
	CP	244 (0.278)	634 (0.722)	0.423	0.916 [0.740–1.135]	30 (0.068)	184 (0.419)	225 (0.513)	0.541	
	Control	226 (0.296)	538 (0.704)			48 (0.089)	243 (0.414)	274 (0.497)		0.888
rs1470612		A	G			A/A	A/G	G/G		
	CP	310 (0.357)	558 (0.643)	0.001^a^	1.408 [1.141–1.738]	63 (0.145)	184 (0.431)	187 (0.431)	0.0009^b^	
	Control	215 (0.283)	545 (0.717)			25 (0.066)	165 (0.434)	190 (0.500)		0.171
rs11706903		A	C			A/A	A/C	C/C		
	CP	297 (0.344)	567 (0.656)	0.010^c^	1.318 [1.067–1.628]	59 (0.137)	179 (0.414)	194 (0.449)	0.017	
	Control	215 (0.284)	541 (0.716)			29 (0.077)	157 (0.415)	192 (0.508)		0.691
rs2606750		A	G		1.128 [0.916–1.390]	A/A	A/G	G/G		
	CP	308 (0.397)	468 (0.603)	0.257		80 (0.206)	148 (0.381)	160 (0.412)	0.207	
	Control	266 (0.368)	456 (0.632)			57 (0.158)	152 (0.421)	152 (0.421)		0.070
rs2594972		T	C			T/T	C/T	C/C		
	CP	398 (0.482)	428 (0.518)	0.0001^d^	1.515 [1.244–1.848]	120 (0.291)	158 (0.383)	135 (0.327)	<0.0001^e^	
	Control	298 (0.380)	486 (0.620)			60 (0.153)1	178 (0.454)	54 (0.393)		0.471
rs4684787		T	C			T/T	C/T	C/C		
	CP	318 (0.360)	566 (0.640)	0.549	0.941 [0.769–1.149]	52 (0.118)	214 (0.484)	176 (0.398)	0.826	
	Control	285 (0.374)	477 (0.626)			49 (0.129)	187 (0.491)	145 (0.381)		0.347

*^*a*^After the SNPSpD correction, the *p*-value is 0.0004; ^*b*^After the SNPSpD correction, the *p*-value is 0.0036; ^*c*^After the SNPSpD correction, the *p-*value is 0.040; ^*d*^After the SNPSpD correction, the *p*-value is 0.0004; ^*e*^After the SNPSpD correction, the *p-*value is < 0.0001. ^∗^Hardy–Weinberg equilibrium.*

**TABLE 6:** Allele and genotype frequencies of *ATG7* in diplegia CP and controls.

**Gene**	**Group**	**Allele frequency**		***p*-Value**	**OR [95% CI]**	**Genotype frequency**			***p*-Value**	**H-W^∗^**
rs346078		C	G			C/C	C/G	G/G		
	CP-Diplegia	53 (0.239)	169 (0.761)	0.066	0.732 [0.524–1.022]	6 (0.054)	41 (0.369)	64 (0.577)	0.176	
	Control	339 (0.300)	791 (0.700)			48 (0.085)	243 (0.430)	274 (0.485)		0.568
rs1470612		A	G			A/A	A/G	G/G		
	CP-Diplegia	86 (0.406)	126 (0.594)	0.0006^a^	1.689 [1.249–2.287]	17 (0.160)	52 (0.491)	37 (0.349)	0.002^b^	
	Control	324 (0.288)	802 (0.712)			43 (0.076)	238 (0.423)	282 (0.501)		0.457
rs11706903		A	C			A/A	A/C	C/C		
	CP-Diplegia	86 (0.398)	130 (0.602)	0.002^c^	1.604 [0.187–2.168]	17 (0.157)	52 (0.481)	39 (0.361)	0.009^d^	
	Control	327 (0.292)	793 (0.708)			50 (0.089)	227 (0.405)	283 (0.505)		0.644
rs2606750		A	G			A/A	A/G	G/G		
	CP-Diplegia	91 (0.369)	109 (0.631)	0.017	1.447 [1.067–1.962]	25 (0.250)	41 (0.410)	34 (0.340)	0.043	
	Control	401 (0.366)	695 (0.634)		1.468 [1.070–2.012]	83 (0.151)	235 (0.429)	230 (0.420)		0.076
rs2594972		T	C			T/T	C/T	C/C		
	CP-Diplegia	112 (0.514)	106 (0.486)	<0.0001^e^	1.718 [1.236–2.387]	32 (0.294)	48 (0.440)	29 (0.266)	0.0015^f^	
	Control	453 (0.383)	729 (0.617)			95 (0.161)	263 (0.445)	233 (0.394)		0.154
rs4684787		T	C			T/T	C/T	C/C		
	CP-Diplegia	63 (0.286)	157 (0.714)	0.012^g^	0.666 [0.486–0.914]	9 (0.082)	45 (0.409)	56 (0.509)	0.025	
	Control	421 (0.376)	699 (0.624)			70 (0.125)	281 (0.502)	209 (0.373)		0.100

*^*a*^After the SNPSpD correction, the *p*-value is 0.0024; ^*b*^After the SNPSpD correction, the *p*-value is 0.008; ^*c*^After the SNPSpD correction, the *p-*value is 0.008; ^*d*^After the SNPSpD correction, the *p-*value is 0.036; ^*e*^After the SNPSpD correction, the *p*-value is < 0.0001; ^*f*^After the SNPSpD correction, the *p*-value is 0.006; ^*g*^After the SNPSpD correction, the *p-*value is 0.048; ^∗^Hardy–Weinberg equilibrium.*

### ATG7 Protein Analysis

**FIGURE 1:**
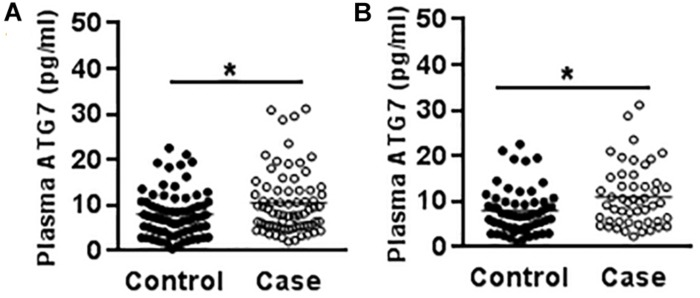
Plasma ATG7 in CP patients and controls. **(A)** The scatter plot of plasma ATG7 levels in CP patients and controls. **(B)** The scatter plot of plasma ATG7 levels in male CP patients and controls. Each dot represents one patient. ^∗^*p* < 0.05; Mann–Whitney *U*-test.

Genetic variants often affect an individual’s susceptibility to disease by affecting the expression level of the encoded protein. The results from a study of 73 cases and 79 controls demonstrated that CP patients showed higher plasma ATG7 levels than the controls (10.58 ± 0.85 vs. 8.18 ± 0.64 ng/ml) (*p* = 0.024) (Figure 1A), and the plasma ATG7 level was higher in male CP patients (11.02 ± 0.94 ng/ml) compared to the male controls (8.28 ± 0.74 ng/ml) (*p* = 0.022) (Figure 1B), but there were no statistical differences in female CP patients compared to female controls, nor any differences regarding spastic/non-spastic CP, term/preterm, with or without periventricular leukomalacia, or with or without maternal premature rupture of membrane.

The genotype-protein expression correlation study indicated that the AA + AG genotype had higher plasma ATG7 protein levels compared to the GG genotype at rs1470612 (8.53 ± 0.73 vs. 7.31 ± 0.56 ng/ml) and that the TT + CT genotype had higher plasma ATG7 levels compared to the CC genotype at rs2594972 (10.54 ± 0.95 vs. 8.53 ± 0.88 ng/ml), but neither of them achieved statistical significance, which might be attributed to the small sample size in the present study.

### Luciferase Reporter Gene Analysis

**FIGURE 2:**
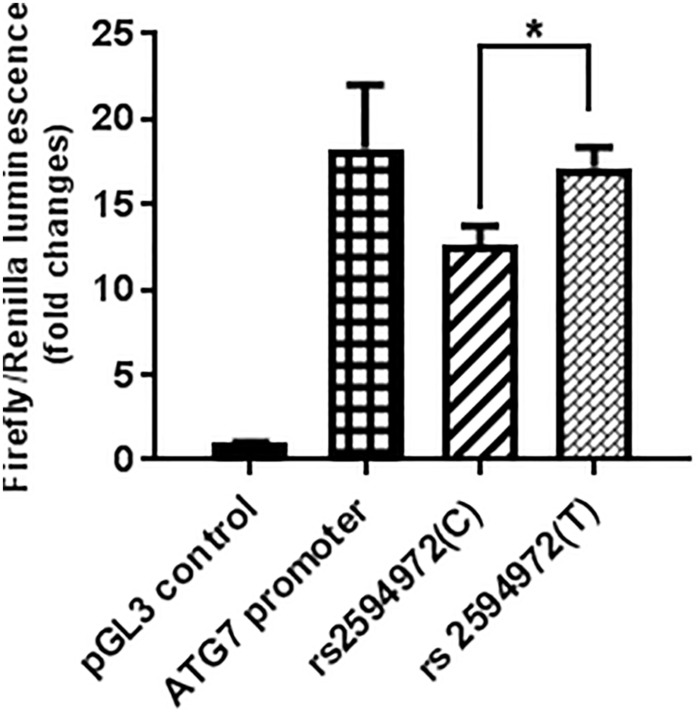
Luciferase reporter assays. The average relative luciferase activity of rs2594972 SNP constructs (T and C), ATG7 promoter, and pGL3 control in 293T cells is indicated in shaded bars, and the standard deviation represented by the error bars (^∗^*p* < 0.01).

The luciferase assay was performed in triplicate (*n* = 3) for each promoter sequence, and a minimal amount of background luciferase activity (0.036 ± 0.002) was measured in 293T cells transfected with control pGL3 luciferase plasmid. The luciferase activity of the pGL3-promoter-rs2594972 mutant construct (T) transfection was significantly increased compared to wild-type (C) transfection in 293T cells (+ 36.3%, *p* = 0.009) (Figure 2). These results indicated that the T allele of rs2594972 might play a specific role in regulation of ATG7 protein expression, and this is consistent with the results of association studies in which the T allele is significantly enriched in the cases.

## Discussion

This case-control study showed that both the allele frequency and genotype frequency of the rs1470612 and rs2594972 SNPs in the *ATG7* gene were associated with CP, and a stronger correlation was found in spastic diplegia and male subgroups, indicating that ATG7 might have an important influence in severe types of CP and that there might be sex-related differences. This study is the first to link *ATG7* genetic variants to CP.

Autophagy is a catabolic recycling pathway conserved from yeast to mammals that uses lysosome degradation to remove damaged organelles and macromolecular material. Maintaining an appropriate level of autophagy activity is important for cell survival, and the loss of autophagic renewal leads to the accumulation of damaged proteins and organelles and subsequent cellular dysfunction. Increasing evidence suggests that autophagy is involved in vertebrate development by modulating various cellular processes, including proliferation, survival, differentiation, and programed cell death (Frudd et al., 2018).

Currently, it has been accepted that autophagy is driven by more than 30 ATG proteins, most of which are involved in the formation of the autophagosome. Among these proteins, ATG7 has long been considered to be an essential molecule for autophagy. In the two ubiquitin-like conjugation systems of microtubule-associated protein light chain 3 (LC3) and ATG12, ATG7, which is essential for the assembly and function of these two conjugates during the expansion of the autophagosomal membrane and the formation of autophagy bubbles and autophagosomes (Mizushima et al., 2011; Feng et al., 2015; Niu et al., 2016). Studies have shown that variants of the *ATG7* gene are related to some diseases, such as sporadic Parkinson’s disease (Chen et al., 2013), systemic lupus erythematous (Dang et al., 2016), acute myocardial infarction (Zhang et al., 2018), and primary ovarian insufficiency (Song et al., 2015). The presence of ATG7 associated with different diseases indicates the pleiotropic effects of ATG7 on many physiological processes and the potentially shared pathological mechanisms of these diseases. So far, no study has shown an association between *ATG7* gene variants and CP. This is the first to focus on the association between *ATG7* gene variants and CP, and we show that CP patients have higher allele A and AA genotype frequencies of rs1470612 and higher allele T and TT genotype frequencies of rs2594972. Haplotype analysis suggested that rs1470612 might coordinate with related sites to play a role in co-regulating ATG7 gene expression in CP.

Additional subgroup analysis showed that the association between the *ATG7* polymorphism at rs1470612 and rs2594972 and CP is stronger in spastic diplegia. Our findings suggest that the presence of the rs1470612 A allele and rs2594972 T allele of the *ATG7* gene significantly increase the risk of CP, and the association of *ATG7* variation with CP appears to be more pronounced in patients with more serious brain injury and disease phenotype.

We further investigated the correlation between *ATG7* gene variants and ATG7 protein expression and found that plasma ATG7 levels were higher in CP patients, which provides further evidence that ATG7 is associated with CP. Furthermore, the level of ATG7 protein expression might be related to the genotypes of rs1470612 and rs2594972. However, there were no statistically significant differences in the correlation analysis between genotype and protein expression, which can very likely be attributed to the limited sample size and inadequate statistical power. Moreover, the luciferase assay confirmed that the T allele of rs2594972, which is enriched in the CP population, significantly increases transcriptional activity of the *ATG7* promoter. These results suggested that the *ATG7* variants might alter the protein level of ATG7 and interfere with autophagy activity. This finding is similar to what is seen in Parkinson’s disease, in which functional studies have shown that the sequence variants within the *ATG7* gene promoter might alter ATG7 protein expression, which would affect autophagy activity and thus contribute as a risk factor to Parkinson’s disease onset (Chen et al., 2013).

The developing brain retains its autophagic machinery to a greater extent than the adult brain in order to facilitate normal brain development (Oppenheim et al., 2008). However, previous experimental studies on the role of autophagy in injury to the developing brain have yielded conflicting results, and both pharmacological inhibition and induction of autophagy have been shown to reduce brain injury after insult (Carloni et al., 2008; Descloux et al., 2018). The confusing results might be related to the limited specificity of autophagy-related drugs (Wu et al., 2010), and studies using a more specific genetic approach have confirmed that autophagy inhibition affords neuroprotection after insult in the developing brain (Koike et al., 2008; Ginet et al., 2014b; Xie et al., 2016). In contrast, increased autophagy activity in the adult brain prevents neurodegenerative disorders (Nixon, 2013; Miki et al., 2018). These results suggest that autophagy plays different roles in the process of neurological disorders between the developing brain and adult brain, and therapeutic strategies targeting autophagy should be adjusted according to developmental stage.

Furthermore, mice deficient in Atg7 have also shown contradictory roles of autophagy. Neuron-specific Atg7 knockout mice exhibit a wide range of neurodegenerative symptoms, including massive loss of neurons in the cerebral and cerebellar cortices, accumulation of inclusion bodies in neurons, and defects of behavioral coordination (Komatsu et al., 2006). The Purkinje cell-specific Atg7 knockout obstructed autophagy-related membrane trafficking and turnover, which led to axonal dystrophy associated with neurodegenerative disease (Komatsu et al., 2007). The CamKII-Atg7^–/–^ mice showed protective roles for Atg7 in neurodegeneration of forebrain neurons, and autophagy deficiency led to reduce amyloid beta (Aβ) secretion and simultaneous accumulation of intracellular Aβ peptide (Inoue et al., 2012; Nilsson et al., 2013). These findings suggest that ATG7-dependent autophagy might protect against the development of many neurological degenerative diseases. Interestingly, the opposite role for ATG7 in dopamine neurons has also been reported. DAT-Atg7^–/–^ mice exhibited an even more serious phenotype of age-dependent neurodegeneration. In contrast, selective deletion of Atg7 in substantia nigra dopaminergic neurons of adult mice produced unexpected protection against retrograde degeneration of dopaminergic axons (Inoue et al., 2012). We also found that selective neural deletion of Atg7 was strongly protective against neural cell death and overall brain injury in a neonatal mouse model of cerebral hypoxia ischemia (Xie et al., 2016). Given that these studies reflect the nature of ATG7’s double-edged sword in development and disease, disturbing the balance of Atg7-mediated autophagy is detrimental.

It has been shown that ATG7 levels in the cerebrospinal fluid were higher in patients with pulmonary encephalopathy, which indicates that ATG7 is associated with brain injury, and experimental studies and clinical sample analyses have shown that autophagy activity is increased in the immature brain after hypoxia ischemia insult and that autophagy inhibition either by genetic knockout of the *Atg7* gene or by pharmacological inhibition provides neuroprotection (Ginet et al., 2014a, b; Xie et al., 2016). We found that plasma ATG7 levels were significantly higher in patients with CP, and this supports our conclusion that deficiency in ATG7-dependent autophagy has a protective role against the development of CP.

Cerebral palsy develops as a result of perinatal or neonatal brain injury, including perinatal hypoxia-ischemia brain injury, preterm white matter injury, and perinatal infection and inflammation (Zhu et al., 2009; Fleiss and Gressens, 2012; Song et al., 2016; Zhang et al., 2017). The initial insult to the immature brain and the injury process can persist for many months or even years (Winerdal et al., 2012), and this suggests that CP might be related to persistent injury processes. Autophagic cell death has been shown to play a critical role in the process of perinatal brain injury (Xie et al., 2016; Descloux et al., 2018), and in this study we found that different alleles and genotypes of the *ATG7* gene SNPs and higher levels of circulating ATG7 protein were significantly associated with CP. We speculate that autophagy might play an important role in persistent brain injury and in the development of CP after the initial insult, and thus the *ATG7* gene might be involved in the pathogenesis of CP. Further studies are needed to determine the dynamic changes of autophagy activity in immature brain injury and to determine the role of epigenetic mechanisms of ATG7 in CP. Moreover, an Atg7-independent type of autophagy (referred to as “alternative autophagy”) has been described (Nishida et al., 2009; Shimizu et al., 2010; Arakawa et al., 2017), and thus Atg7 might be involved in the pathogenesis of CP through an autophagy-independent signaling pathway despite being an autophagy gene.

This is the first study we are aware of showing the details of the association between the *ATG7* gene and CP, and we have shown that variants of the *ATG7* gene contribute to susceptibility to CP by analyzing six SNPs in a relative large number of cases and controls. We have also shown the correlation of genotype-protein expression in the different variants by measuring the plasma level of the ATG7 protein and performing the luciferase analysis. However, there are some limitations in this study. First, the heterogeneity of the case group might have influenced the genetic analysis results. Second, even though the ATG7 protein level in the plasma was analyzed in patients, not every patient was possible for the ATG7 protein assay, which might have influenced the genotype-protein expression correlation analysis. Third, the rare SNPs were not investigated in this study, and they might have had significant impacts on the individual susceptibility to CP.

In summary, we found significant associations between SNPs in *ATG7* with CP in Han Chinese infants. The variants might affect ATG7 expression and autophagy activity and thus might induce persistent brain injury. Thus, autophagy inhibition might be a preventive or therapeutic strategy for CP, and further work should focus on the mechanism of CP induced by autophagy.

## Data Availability Statement

The datasets for this manuscript are not publicly available because it is possible on reasonable request. Request to access the datasets should be directed to changlian.zhu@neuro.gu.se.

## Ethics Statement

Approval for this study was obtained from the Ethics Committee of Zhengzhou University (201201002).

## Author Contributions

LX, JX, JS, YX, DB, YQ, YW, and XZ performed the experiments and analyzed the data. LX, JX, XW, QX, and CLZ wrote the manuscript. JS, YX, BZ, DZ, CG, CCZ, XZ, QS, and LX provided the samples. CLZ, QX, and XW designed the study and revised the manuscript. All authors read and approved the final manuscript.

## Conflict of Interest

The authors declare that the research was conducted in the absence of any commercial or financial relationships that could be construed as a potential conflict of interest.

## Footnotes

1 www.ncbi.nlm.nih.gov/SNP

2 http://snpinfo.niehs.nih.gov/

3 http://www.analysis.bio-x.cn/
